# Identification of a Novel Gig2 Gene Family Specific to Non-Amniote Vertebrates

**DOI:** 10.1371/journal.pone.0060588

**Published:** 2013-04-04

**Authors:** Yi-Bing Zhang, Ting-Kai Liu, Jun Jiang, Jun Shi, Ying Liu, Shun Li, Jian-Fang Gui

**Affiliations:** State Key Laboratory of Freshwater Ecology and Biotechnology, Institute of Hydrobiology, Chinese Academy of Sciences, Wuhan, China; INRA, France

## Abstract

*Gig2* (grass carp reovirus (GCRV)-induced gene 2) is first identified as a novel fish interferon (IFN)-stimulated gene (ISG). Overexpression of a zebrafish *Gig2* gene can protect cultured fish cells from virus infection. In the present study, we identify a novel gene family that is comprised of genes homologous to the previously characterized *Gig2*. EST/GSS search and in silico cloning identify 190 *Gig2* homologous genes in 51 vertebrate species ranged from lampreys to amphibians. Further large-scale search of vertebrate and invertebrate genome databases indicate that *Gig2* gene family is specific to non-amniotes including lampreys, sharks/rays, ray-finned fishes and amphibians. Phylogenetic analysis and synteny analysis reveal lineage-specific expansion of *Gig2* gene family and also provide valuable evidence for the fish-specific genome duplication (FSGD) hypothesis. Although Gig2 family proteins exhibit no significant sequence similarity to any known proteins, a typical Gig2 protein appears to consist of two conserved parts: an N-terminus that bears very low homology to the catalytic domains of poly(ADP-ribose) polymerases (PARPs), and a novel C-terminal domain that is unique to this gene family. Expression profiling of zebrafish *Gig2* family genes shows that some duplicate pairs have diverged in function *via* acquisition of novel spatial and/or temporal expression under stresses. The specificity of this gene family to non-amniotes might contribute to a large extent to distinct physiology in non-amniote vertebrates.

## Introduction

A gene family is composed of homologous genes that are formed by duplication of a single original gene. Duplication events can be classified into two types according to the mechanisms of their origins: whole genome duplication (WGD) and segmental duplication (SD) [1], both of which provide primary resources for the origin of gene families. Two rounds of WGDs are hypothesized to have occurred on the jawed vertebrate stem, after the divergence of urochordates but before the split between cartilaginous fish and bony vertebrates [2]; teleost fish is suggested to have specifically undergone a third round of WGD termed as fish-specific genome duplication (FSGD) [3]. SD is believed to contribute a lot to lineage-specific expansion of gene families [4], while WGD is important for shaping diversity and complexity of modern vertebrates, because reciprocal gene loss and re-diploidization after WGDs might contribute to the radiation of vertebrates [5].

The members of a gene family are likely to have highly similar functions, such as vertebrate interferon (IFN) family genes, all of which share a common ability to block cellular replication of different viruses [6]. In mammals, viral infection results in activation of an innate immune response for establishment of a host antiviral state, which is characterized by the production of IFN family proteins and the subsequent transcriptional upregulation of IFN-stimulated genes (ISGs) [7]. Dozens of gene families are involved in IFN antiviral immune response. For example, Toll-like receptor (TLR) family members (such as TLR3/4/7/8/9) and retinoic acid-inducible gene I (RIG-I)-like receptor (RLR) family members recognize different pathogen-associated molecular patterns (PAMPs) to trigger type I IFN signalling [6], [7]; IFN-regulatory factor (IRF) 3 and IRF7, two IRF family members, directly control expression of IFN family genes [7], [8], [9]; IFIT (IFN-induced proteins with Tetratricopeptide Repeat (TPR) motifs) family members mediate IFN antiviral effects by selectively restricting the replication of virus lacking 2′-O methylation mRNA or with a 5′-triphosphate RNA [10], [11]. Recently a new strategy has been proposed, in which cells reduce sensitivity to viruses through modification of the viral proteins by poly(ADP-ribosyl)ation (PARylation), a post-translational modification of proteins that is catalyzed by poly(ADP-ribose) polymerases (PARPs) [12].

Similar to mammals, fish possess conserved IFN antiviral response by the TLR pathway and the RLR pathway [6], [13]. Consistently, fish contain almost all subsets of TLRs corresponding to human TLRs [14]; the same is true for RLR family members [15]. Fish IRF family members show a clear orthologous relationship with mammalian counterparts [16]. In addition, fish IFNs exert antiviral function by induction of an array of ISGs that are conserved in fish [8], [17], [18]. However, an attempt to screen fish IFN responsive genes has identified some novel ISGs including *Gig2* (grass carp reovirus (GCRV)-induced gene 2) [19], [20]. Interestingly, *Gig2* is not significantly homologous to any known genes and seems not to be found in mammalian genomes [19], which has sparked great interests to investigate its evolutionary origin.

Although there is low or no constitutive expression of crucian carp *Carassius auratus Gig2* in cultured fish cells [19], it can be detected in tissues of healthy grass carp (*Ctenopharyngodon idella*) at mRNA and protein levels [21]. Despite these differences, Gig2 mRNA and protein are highly up-regulated by viral infection, IFN and poly (I:C) treatment [19], [20], [21]. Actually, *Gig2* is a typical ISG [21] and is activated by fish IFN signalling [8], [22], [23]. Overexpression of a zebrafish *Gig2* member (*DreGig2I*) prevents spring viremia of carp virus (SVCV) and *Rana grylio* virus (RGV) infection in EPC (Epithelioma papulosum cyprinid) cells [23]. This result confirms a potential antiviral role of fish Gig2, although the detailed mechanisms remain elusive. Strikingly, at least two proteins homologous to crucian carp Gig2 are found in IFN-produced fish cells [21], giving a clue that there may be a gene family homologous to *Gig2* gene.

In the present study, we reported identification of a novel gene family termed *Gig2* gene family. Strikingly, *Gig2* gene family is specific to non-amniotes including lampreys, sharks/rays, ray-finned fishes and amphibians, by large-scale search of EST, GSS and genome databases. Phylogenetic analyses revealed an extensive expansion of *Gig2* gene family by lineage-specific and species-specific duplication. Expression characterization of zebrafish *Gig2* family genes suggested that some duplicate pairs had diverged in function. In spite of no significant homology to known proteins, a typical Gig2 protein is composed of an N-terminus that exhibits very low homology to PARP catalytic domain and a C-terminus that is conserved in all Gig2 proteins but no homology to any a known domain.

## Results

### Identification of *Gig2* Genes in Crucian Carp

**Figure 1:**
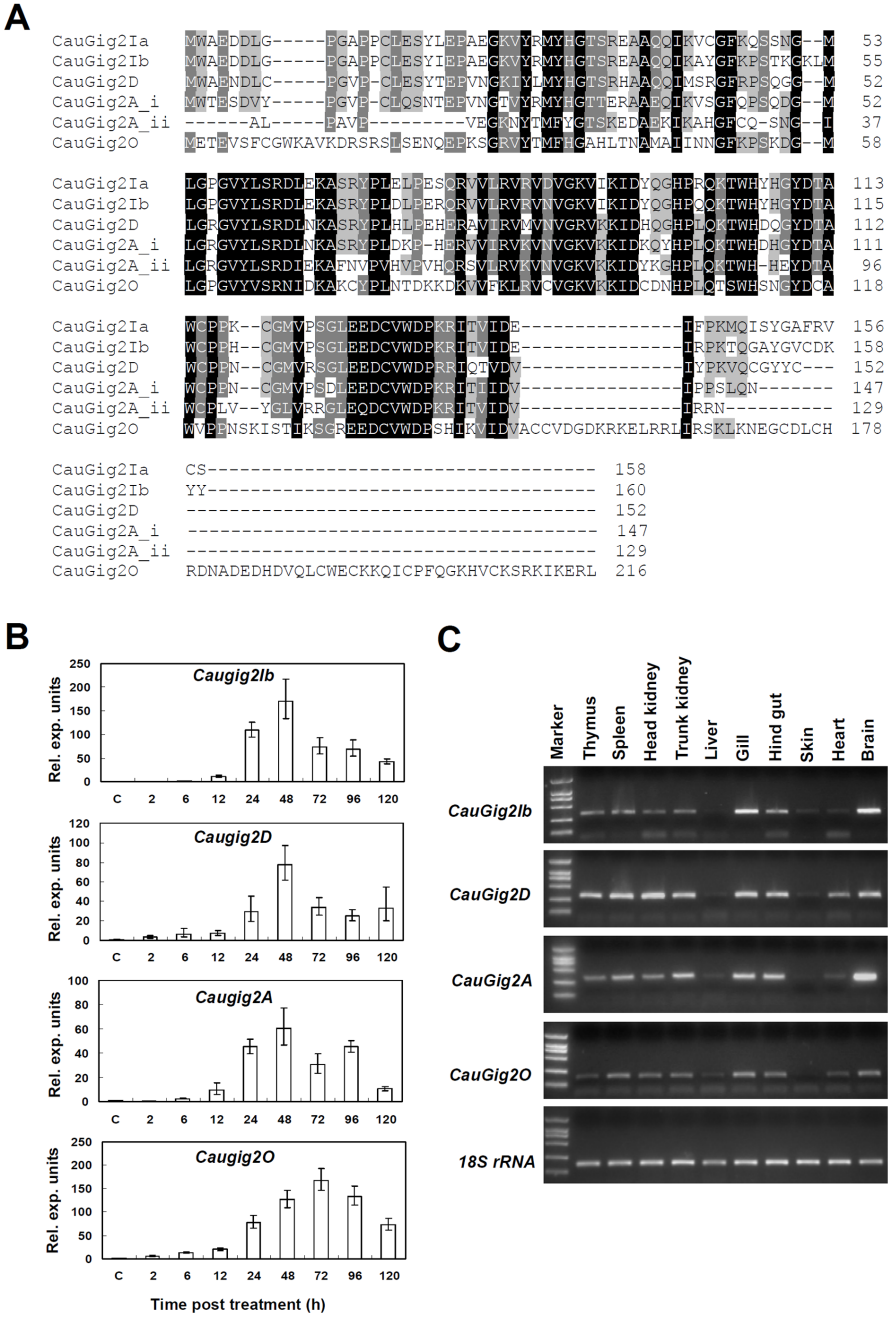
Identification of *Gig2* gene family in crucian carp. (A) Multiple alignments of five crucian carp Gig2 proteins were performed by ClustalW. Identical amino acid residues are highlighted with black shading, while gray shading was used for regions with more than 60% identity, or where more than 9 of 14 amino acid residues shared the same properties. (B) Induction of crucian carp *Gig2* genes by UV-inactivated GCRV (1×10^9^ TCID50 ml/L exposed to UV irradiation). CAB cells were treated with UV-inactivated GCRV for 2, 6, 12, 24, 48, 72, 96 and 120h, respectively. Real-time PCR was used to detect transcripts of crucian carp *Gig2* genes. The ratio of *Gig2s* to β-actin in control cells was set to 1, and all treated cells were normalized relative to this value. Error bars represent standard deviations obtained by measuring each sample three times. (C) RT-PCR detection of *Gig2* transcripts in indicated tissues from healthy grass carps. 18S rRNA was used as the endogenous control.

Sequencing of 2035 ESTs from a subtractive cDNA library, which was made by UV-inactivated GCRV-infected *Carassius auratus* blastulae embryonic cells (CAB) against mock-infected CAB cells [24], retrieved 45 ESTs, which were homologous to a previously identified gene, *Gig2* (termed *CauGig2Ia* in this paper) [19]. These ESTs were assembled to 4 contigs, according to which 4 full-length cDNAs besides *CauGig2Ia* were obtained by RACE-PCR. These 4 genes were subsequently termed *CauGig2A, CauGig2D, CauGig2Ib* and *CauGig2O* with reference to the locus of zebrafish *Danio rerio* chromosome that was described thereafter. Multiple alignments reveal a high level of amino acid sequence identity between them, from 40% (CauGig2A verse CauGig2O) to 87% (CauGig2Ia verse CauGig2Ib) (Fig. 1A). Strikingly, CauGig2A contains 276 amino acids, almost twice in length than the other four proteins. Actually, CauGig2A is composed of two homologues units, since the 147-amino-acid-N terminus and the 129-amino-acid-C terminus of CauGig2A display a high level of sequence identity either to each other or to the other four proteins (Fig. 1A).

Similar to *CauGig2Ia* that was upregulated by GCRV infection and fish IFN [21], the other four genes were transcriptionally induced in CAB cells treated with UV-inactivated GCRV, exhibiting a similar expression pattern where the transcription expression gradually increased with stimulated time and reached a peak and then decreased, although the peak of *CauGig2O* was later than others (Fig. 1B). Additionally, these genes showed a ubiquitous and constitutive expression in tissues of healthy grass carps, with a relatively high level of transcription in thymus, spleen, head kidney, trunk kidney, gill, hindgut and brain, but a low level of transcription in liver, skin and heart (Fig. 1C), which was similar to *CauGig2Ia* [21].

### Identification of *Gig2* Gene Clusters in Zebrafish

**Figure 2:**
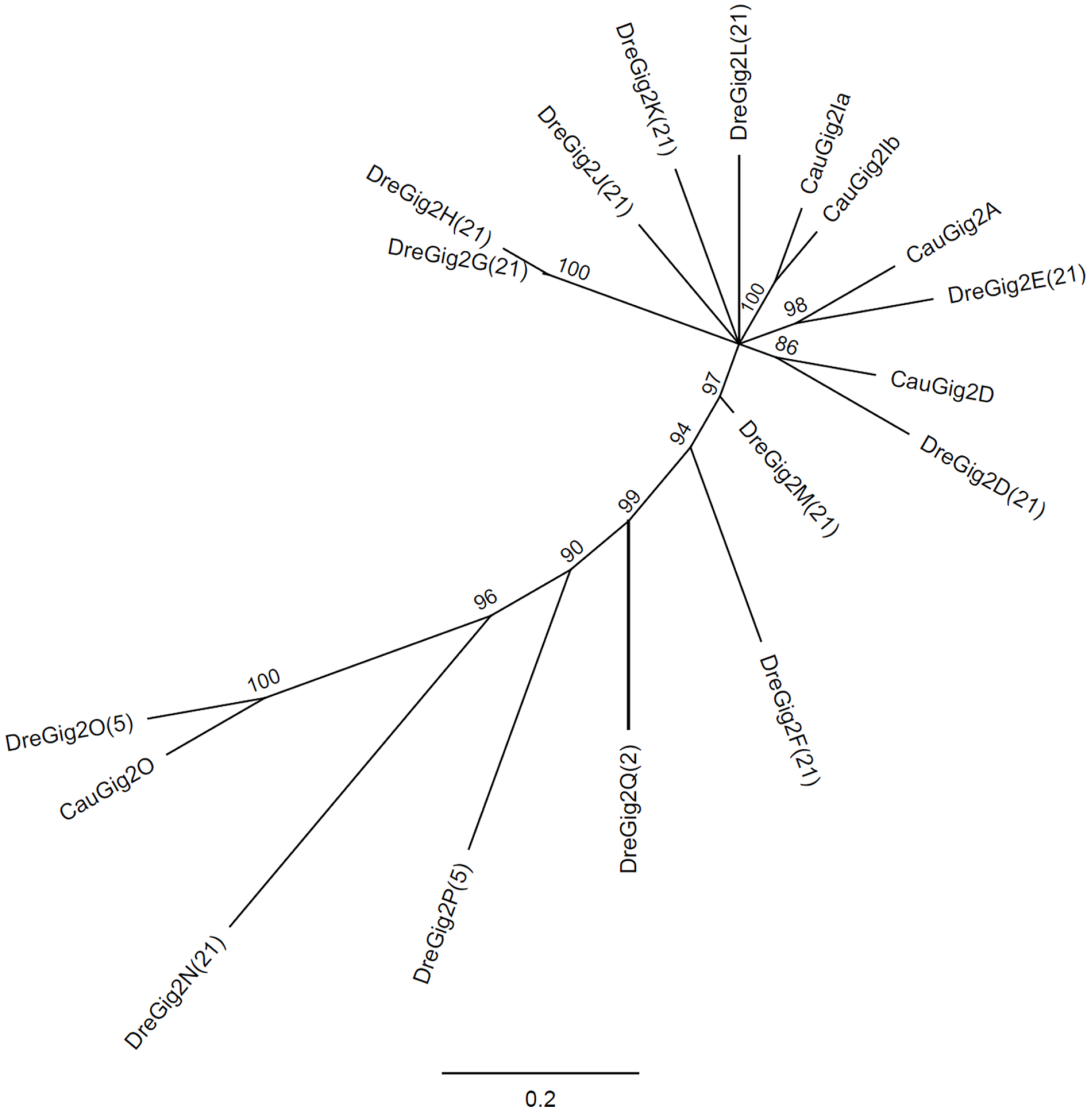
Phylogenetic tree of zebrafish and crucian carp Gig2 family. A neighbour-joining tree was constructed based on analyses of 14 zebrafish Gig2 protein sequences and 5 crucian carp Gig2 protein sequences by the software Geneious using the Neighbour-Joining method with a bootstrap test of 1000 replicates. The accession numbers and sequences of Gig2 family members are shown in Table S1.

Simultaneous responsiveness of multiple crucian carp *Gig2* homologous genes to viral infection sparked our interests to investigate whether there was a *Gig2* gene family in fish genomes. Initially, we searched zebrafish genome using crucian carp Gig2 protein sequences as queries by tBLASTn. 20 orthologous genes were found in zebrafish genome of the version 7 (Zv7), termed *DreGig2A* to *DreGig2T*. However, only 13 homologous genes were retained in zebrafish genome of Zv9, all of which were distributed in three gene loci: one locating on chromosome 21 with 10 members (*DreGig2D* to *DreGig2N* but lacking *DreGig2I*), one on chromosome 5 with 2 members (*DreGig2O* and *DreGig2P*), and one on chromosome 2 with only one member (*DreGig2Q*). *DreGig2M* was assumed to be a pseudogene according to genome annotation. Phylogenetic analysis revealed an orthologous relationship between zebrafish genes and crucian carp counterparts, indicating that *Gig2* gene family in zebrafish and crucian carp had extensively expanded at least before the split of two fish species (Fig. 2).

**Figure 3:**
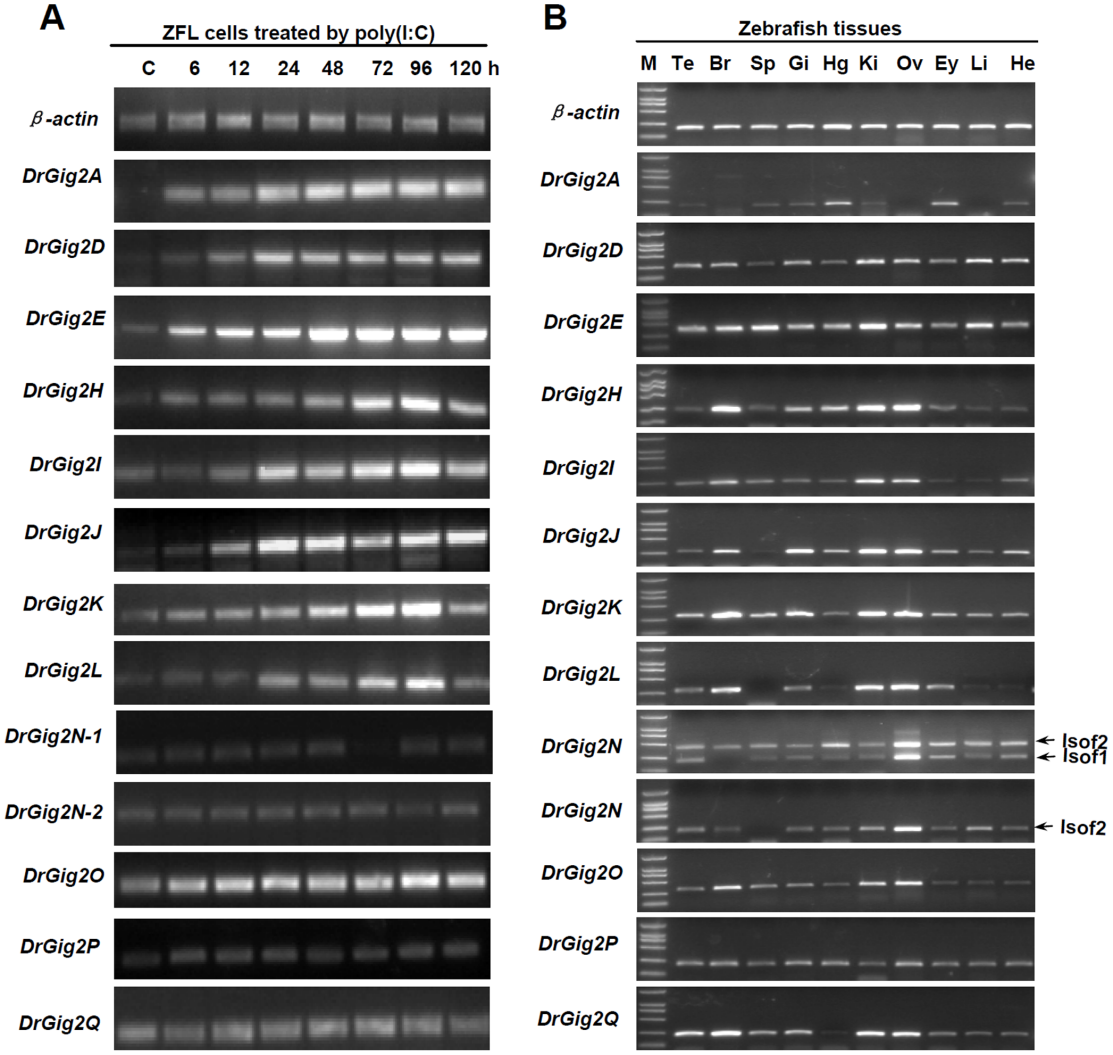
Expression characterization of zebrafish *Gig2* genes. (A) ZFL cells seeded on 6-well plates overnight were transfected with 2 µg/ml poly(I:C) for 6, 12, 24, 48, 72, 96, and 120 h. Then total RNAs were extracted to examine the expression level of zebrafish *Gig2* transcripts by RT-PCR. (B) RT-PCR detection of *Gig2* transcripts in the indicated tissues from healthy zebrafish. β-actin was used as the endogenous control. Isof1 and Isof2 refer to the two isoforms of zebrafish *Gig2N*. All products of RT-PCR were checked by sequencing. M-Marker, Te-Testis, Br-Brain, Sp-Spleen, Gi-Gills, Hg-Hindgut, Ki-kidney, Ov-Ovary, Ey-Eye, Li-Liver, He-Heart.

Subsequently, the expression profiles of zebrafish *Gig2* genes were investigated. Most genes were significantly upregulated in ZFL cells when transfected with poly(I:C), an effective IFN inducer [8], but four (*DreGig2N, O, P, Q*) not (Fig. 3A). *DreGig2N* had two different isoforms with the same ORF which might be caused by alternative splicing. Consistently, two PCR bands were detected by specific primers (Fig. 3A). In addition, zebrafish *Gig2* genes were expressed ubiquitously in all tissues tested, although at differential expression levels (Fig. 3B). Notably, we failed to detect the expression of *DreGig2F* by RT-PCR. Although both *DreGig2A* and *DreGig2I* were not found in zebrafish genome of Zv9, their transcripts were detected *in vitro* and *in vivo* (Fig. 3). These data indicated that there existed a Gig2 gene family in zebrafish, the members of which seemed to have diverged in biological function.

### Specificity of *Gig2* Gene Family to Non-amniotes

To investigate the distribution of *Gig2* gene family in different species, we searched the genome sequences in the databases of ensembl and NCBI. 23 sequences orthologous to *Gig2* were found from stickleback *Gasterosteus aculeatus*, medaka *Oryzias latipes*, green spotted pufferfish *Tetraodon fluviatilis*, takifugu *Takifugu rubripes* and frog *Xenopus laevis* in addition to zebrafish (Table 1). Then we searched non-redundant nucleotide databases of NCBI, and 9 *Gig2* cDNAs were specifically hit besides crucian carp and zebrafish (Table S1). No additional protein sequences other than the sequences encoded by the identified cDNAs were found. To further investigate the species distribution of *Gig2* gene family, we screened EST and GSS databases of NCBI by BLASTN using all identified *Gig2* cDNA sequences as probes. 870 ESTs and 22 GSS were hit in 51 vertebrate species ranged from lampreys to amphibians (Table 1, Table S2). Unexpectedly, 3 ESTs in two algae and 5 GSSs in marine metagenomes were found encoding two *Gig2*-like genes in algae and 5 genes in marine water samples (Table S2), but we think that they might be caused by horizontal gene transfer [25]. We then assembled the *Gig2* ESTs and GSSs using the CAP3 online service, and 140 cDNAs with putative full ORFs were *in silico* cloned and identified (Table S1). Together with the cDNAs and sequences from genomes, a total of 190 *Gig2* genes were identified and distributed in 53 species including 2 algae (Table 1). Consistent with a taxonomy common tree constructed with the 53 species and other 10 representative species (Fig. S1), the identified *Gig2* genes were found to be specific to non-amniote vertebrates, widely in ray-finned fishes (Table 1, Fig. S1). To exclude the possibility of eluding from the lower or upper species, we searched all the available databases of *Caenorhabditis elegans* (roundworm), *Drosophila melanogaser* (fruit fly), *Strongylocentrotus purpuralus* (sea urchin), *Branchiostoma lanceolatum* (lancelet), *Anolis carolinensis* (lizard), *Gallus gallus* (chicken), *Mus musculus* (mouse) and *Homo sapiens* (human), but no *Gig2* homologous sequences were found. Therefore, *Gig2* gene family is specific to non-amniotes including lampreys, sharks/rays, ray-finned fish and amphibians regardless of the two algae.

**Table 1:** Summary of Gig2 genes identified in this study.

category	organism	Sequence sources	No.
ray-finned fish	sablefish *Anoplopoma fimbria*	28 EST	2
	white sturgeon *Acipenser transmontanus*	8 EST/GSS	1
	Crucian carp *Carassius auratus*	5 complete mRNA	5
	common carp *Cyprinus carpio*	10 EST	3
	lake whitefish *Coregonus clupeaformis*	4 EST	1
	European seabass *Dicentrarchus labrax*	8 EST/GSS	6
	Antarctic toothfish *Dissostichus mawsoni*	2 GSS	1
	zebrafish *Danio rerio*	133EST, Genome (Chr.1,5,21)	13
	northern pike *Esox lucius*	15 EST	3
	mummichog *Fundulus heteroclitus*	4 EST	2
	stickleback *Gasterosteus aculeatus*	2EST; Genome (Chr. XI, XIII)	4
	Atlantic cod *Gadus morhua*	20 EST	6
	Atlantic halibut *Hippoglossus hippoglossus*	2 EST	1
	blue catfish *Ictalurus furcatus*	10 EST	5
	channel catfish *Ictalurus punctatus*	99 EST/GSS	10
	Japanese seabass *Lateolabrax japonicus*	1 EST	1
	Barramundi perch *Lates calcarifer*	1 EST	1
	*Lipochromis sp. 'matumbi hunter'*	5 EST	2
	Oriental weatherfish *Misgurnus anguillicaudatus*	177 EST	10
	Brown croaker *Miichthys miiuy*	2 EST	1
	Japanese medaka *Oryzias latipes*	6 GSS; Genome (Chr.8, Chr.9)	4
	Rainbow smelt *Osmerus mordax*	1 EST	1
	Rainbow trout *Oncorhynchus mykiss*	35 EST	9
	Nile tilapia *Oreochromis niloticus*	13 EST/GSS, Genome	4
	turbot *Psetta maxima*	2 EST	1
	Japanese flounder *Paralichthys olivaceus*	1 EST	1
	Fathead minnow *Pimephales promelas*	2 EST	1
	guppy *Poecilia reticulata*	1 EST	1
	*Ptyochromis sp.'redtail sheller'*	3 EST	2
	Roach minnow *Rutilus rutilus*	1 EST	1
	gilthead seabream *Sparus aurata*	19 EST	5
	copper rockfish *Sebastes caurinus*	9 EST	4
	Chinese perch *Siniperca chuatsic*	1 complete mRNA	1
	brook trout *Salvelinus fontinalis*	8 EST	3
	grass rockfish *Sebastes rastrelliger*	2 EST	1
	Atlantic salmon *Salmo salar*	167 EST	16
	green pufferfish *Tetraodon fluviatilis*	1 EST	1
	green spotted pufferfish *Tetraodon nigroviridis*	9 EST, Genome (Chr.3, 12)	10
	fugu *Takifugu rubripes*	3 EST, Genome (Sf705, 101)	4
	Graying *Thymallus thymallus*	3 EST	1
	grass goby *Zosterisessor ophiocephalus*	1 EST	1
Sharks/rays	little skate *Leucoraja erinacea*	1 EST	1
	spiny dogfish *Squalus acanthias*	2 EST	1
Frogs/toads	African clawed frog *Xenopus laevis*	16 EST/GSS	8
	Silurana *Xenopus (Silurana) tropicalis*	Genome	5
salamanders	Axolotl *Ambystoma mexicanum*	12 EST	5
	Eastern tiger salamander *Ambystoma tigrinum tigrinum*	18 EST	5
	Japanese firebelly newt *Cynops pyrrhogaster*	8 EST	3
	Eastern newt *Notophthalmus viridescens*	12 EST	2
lampreys	*Lethenteron japonicum*	1 EST	1
	sea lamprey *Petromyzon marinus*	7 EST	2
dinoflagellates	*Karenia brevis*	2 EST	1
haptophytes	*Emiliania huxleyi*	1 EST	1
marine genome	Marine metagenome water samples	5 EST/GSS	5

### Domain Analyses of the Putative Gig2 Proteins

**Figure 4:**
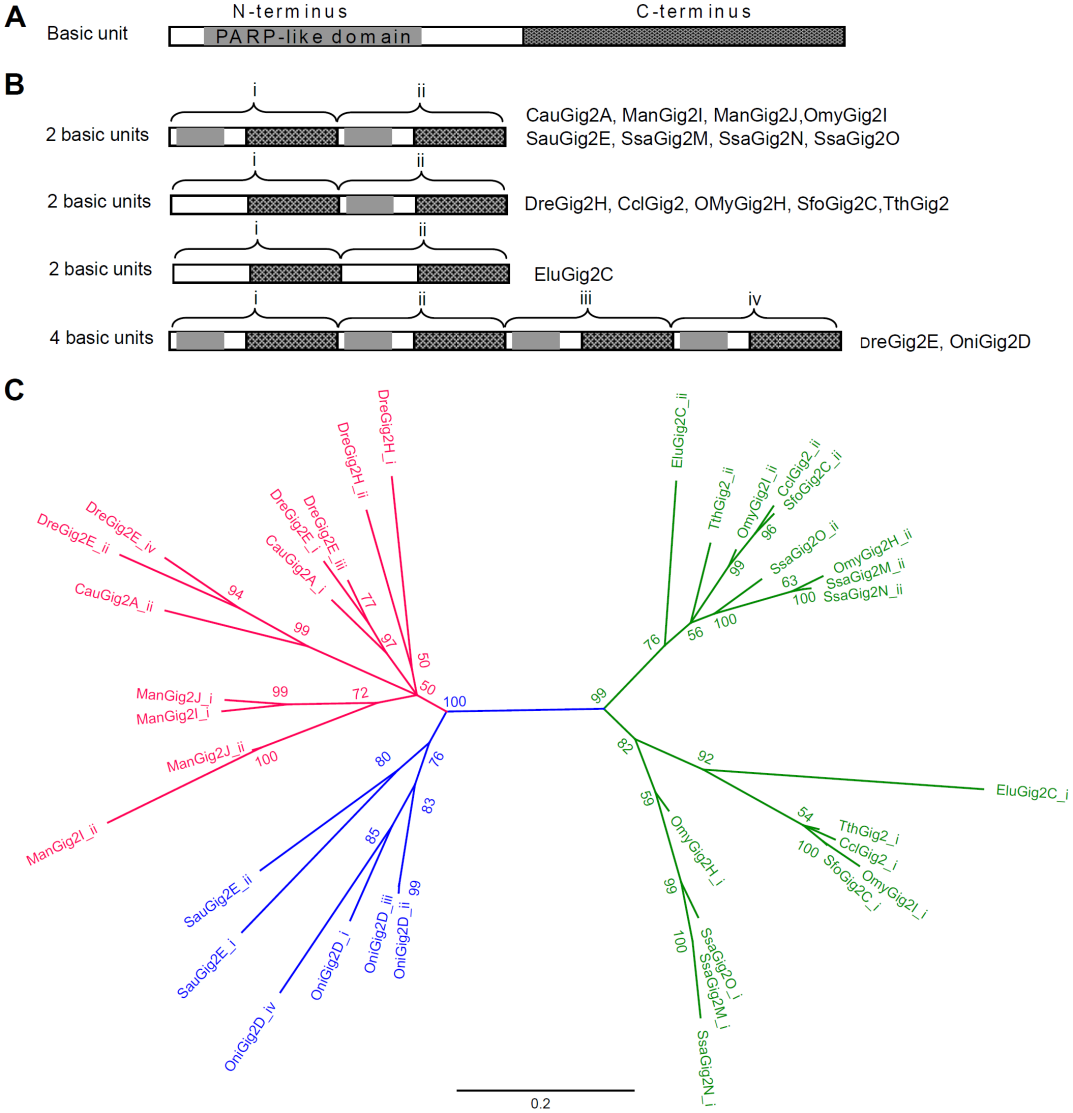
Domain analyses of Gig2 family proteins. (A) Schematic diagram of the structure of a typical Gig2 protein (Basic unit), with an N-terminus containing a stretch weakly homologous to PARK-like domain and a conserved C-terminus. (B) Schematic diagram of Gig2 proteins with two tandem units or four tandem units. (C) Phylogenetic tree of separate units of Gig2 proteins with two or four tandem units by the software Geneious using the Neighbour-Joining method with a bootstrap test of 1000 replicates. Sequences containing 2 basic units or 4 basic units were cut into 2 parts or 4 parts, which was suffixed by “_i” and “_ii” or by “_i”, “_ii”, “_iii” and “_iv”, respectively.

To determine whether there existed any known protein domains, we searched NCBI's conserved domain databases using each putative Gig2 protein sequence. 165 out of 190 Gig2 protein sequences hit a domain named Pssmid 30070 (TCCD_inducible_PARP_like) with a very low bitscore (generally less than 50, most with 40 or so) (Table S1). Further searching Pfam-A (http://pfam.sanger.ac.uk/) found that Pssmid 30070 (TCCD_inducible_PARP_like) is a PARP catalytic domain with about 230 residues in length, the active site of which is composed of a block of 50 amino acids, known as the 'PARP signature' [26], [27]. Multiple alignments showed that a partial N-terminal stretch of all Gig2 proteins is weekly homologous to the region equivalent to the PARP signature (Table S1), even in the Gig2 members that were not hit to the Pssmid 30070 by NCBI batch CD search (data not shown). In addition, there is high sequence homology between the C-terminus of Gig2 proteins although no known domains were predicated. Accordingly, a typical Gig2 protein might contain two conserved parts, one N-terminus with a stretch weekly homologous to the PARP signature, and one C-terminus with no significant homology to any a known domain (Fig. 4A).

Interestingly, some Gig2 proteins appear to have two stretches that weakly hit Pssmid 30070 (PARP-like domain), including CauGig2A, ManGig2I, ManGig2J, OmyGig2I, SauGig2E, SsaGig2M, SsaGig2N and SsaGig2O (Table S1). Similar to CauGig2A in Fig. 1A, they are composed of 2 tandem basic units with a high level of homology (data not shown), each of which has one conserved N-terminus and one conserved C-terminus with reference to the typical Gig2 protein (containing 1 basic unit) (Fig. 4B). The former part was named by appending "_i" to the gene name to differentiate the latter part appended by "_ii", and both parts exhibited highly homology (Fig. 4B). 2 tandem units were also seen in DreGig2H, CclGig2, OMyGig2H, SfoGig2C, TthGig2, in which the N-terminus in the latter unit was weakly hit on Pssmid 30070, and EluGig2C with no hits on Pssmid 30070. Strikingly, DreGig2E and OniGig2D were found to consist of 4 tandem units, and therefore cut into 4 parts: i, ii, iii and iv (Fig. 4B).

To further determine the origin of proteins with multiple units, the separated basic unit sequences were subjected to construct a N-J phylogenetic tree. Four major clades were formed. One was comprised of fish sequences from the order *Cypriniformes* (the red in Fig. 4C), two from the order *Salmoniformes* (the green in Fig. 4C), and one from the order *Pseudocrenilabrinae* (the blue in Fig. 4C). This result indicated that domain duplications of these Gig2 proteins had occurred after the split of the ancestors of *Salmoniformes*, *Cypriniformes,* and *Pseudocrenilabrinae*. In a specific order group, the former moieties of sequences with two tandem units were likely to cluster together, and the same was true to the latter parts (Fig. 4C). For DreGig2E, the unit_i was clustered with the unit_iii, and the unit_ii with the unit_iv; for OniGig2D, the unit_i was clustered with the unit_iv, and the unit_ii with unit_iii(Fig. 4C), suggesting that both four-unit-containing proteins were generated by internal duplication of a two-unit-containing protein.

### Lineage-specific Gene Duplication of *Gig2* Gene Family

**Figure 5:**
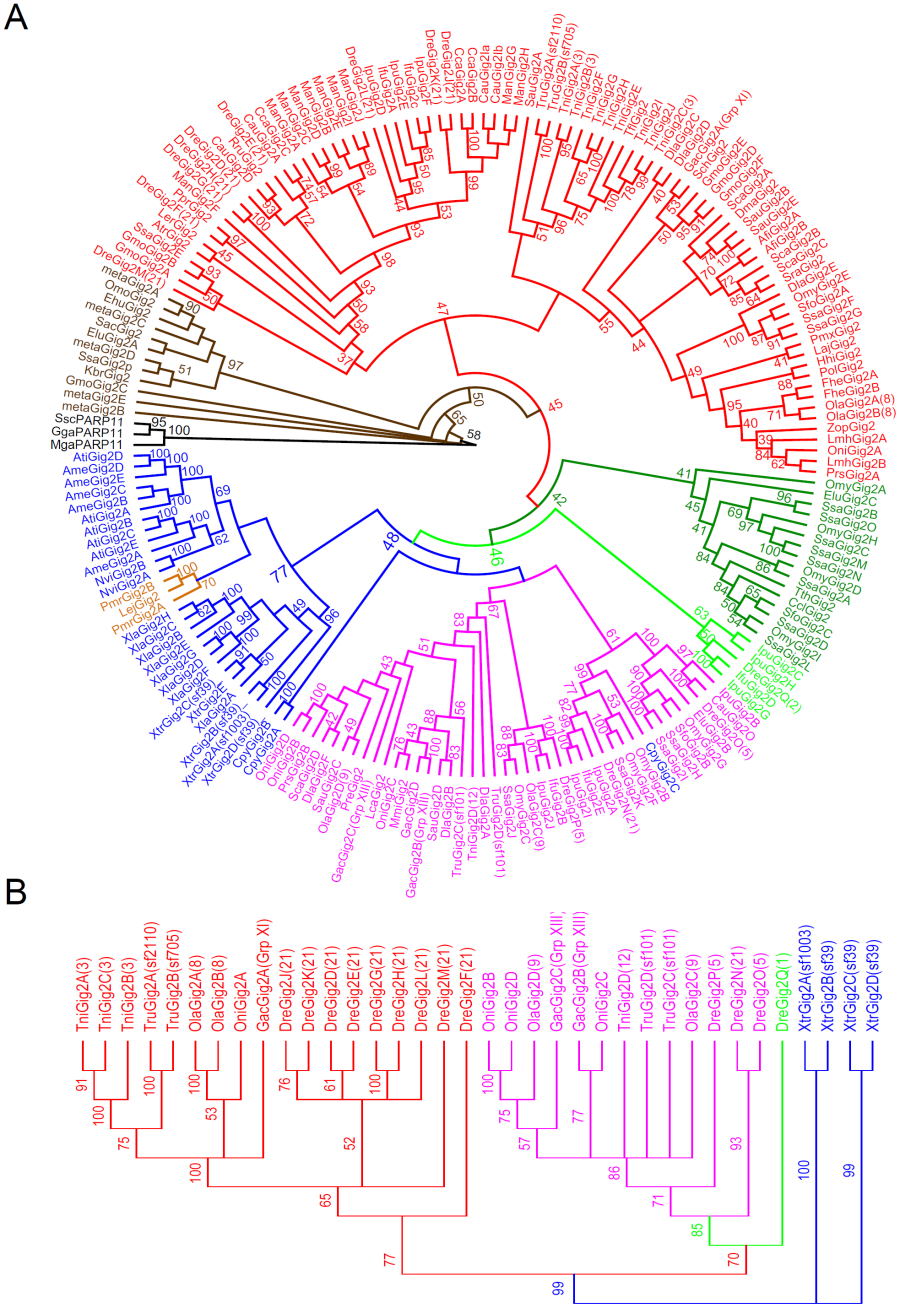
Phylogenetic analyses of Gig2 family proteins. (A) Phylogenetic analysis of all Gig2 proteins identified in this study. A neighbour-joining tree was constructed based on analysis of 190 Gig2 family protein sequences using Geneious, with bootstrap values for 1000 replicates. Three PARP11 proteins were included as an outgroup. The evolutionary distances were computed using the Poisson correction method with the pairwise deletion option. (B) Phylogenetic analyses of Gig2 proteins with definite chromosomal information. The indication by different colours was seen in the text.

Phylogenetic trees were constructed to investigate the evolutionary history of 190 Gig2 family proteins identified above. Since Gig2 proteins exhibited weak homology to PARP family members, three PARP 11 proteins from chicken, turkey and pig were included as an outgroup. As shown in Fig. 5A, Gig2 members were clustered into six major clades, except that 12 Gig2 members mainly from marine water samples and algae showed some distant relationship to these six groups (the dark brown in Fig. 5A). Most amphibian Gig2 family members were clustered into one clade (the blue in Fig. 5A), whereas fish Gig2 members were clustered into four major clades. Three lamprey Gig2 members were grouped with amphibian-related clade (the light brown in Fig. 5A), which was possibly ascribed to a rapider evolution during radiation of teleost species than was seen in lamprey and amphibian lineages [28]. Notably, Gig2 members from the close species were more likely to cluster together. This was consistent with the evolutionary relationship indicated by the taxonomy common tree (Fig. S1). For example, Gig2 members from two frogs were clustered in one subgroup, whereas those from four salamanders clustered in another (the blue in Fig. 5A). The same was true for four major fish-specific clades. These results suggested that Gig2 gene family had been extensively expanded by lineage-specific duplication.

In addition, the major four fish-specific clades were clustered dependently on chromosome locations. Specifically, while Gig2 members from zebrafish chromosome 21, stickleback chromosome group XI, medaka chromosome 8 and green spotted prufferfish chromosome 3 were clustered in one clade (the red in Fig. 5A), the members from zebrafish chromosome 5, stickleback chromosome group XIII, medaka chromosome 9 and green spotted pufferfish chromosome 12 were clustered in another one (the pink in Fig. 5A). However, DreGig2Q on zebrafish chromosome 2 and the other four *Cypriniformes* members were grouped to form one different clade (the light green in Fig. 5A); there was an additional *Salmoniformes*-related clade that consisted of sequences without definite chromosome information (the dark green in Fig. 5A). Further, 36 Gig2 members with definite chromosome information were subjected to make an unrooted phylogenetic tree. Similar to the findings above, these genes located on two sets of fish chromosomes were clearly grouped into two major clades (the red and the pink in Fig. 5B).

**Figure 6:**
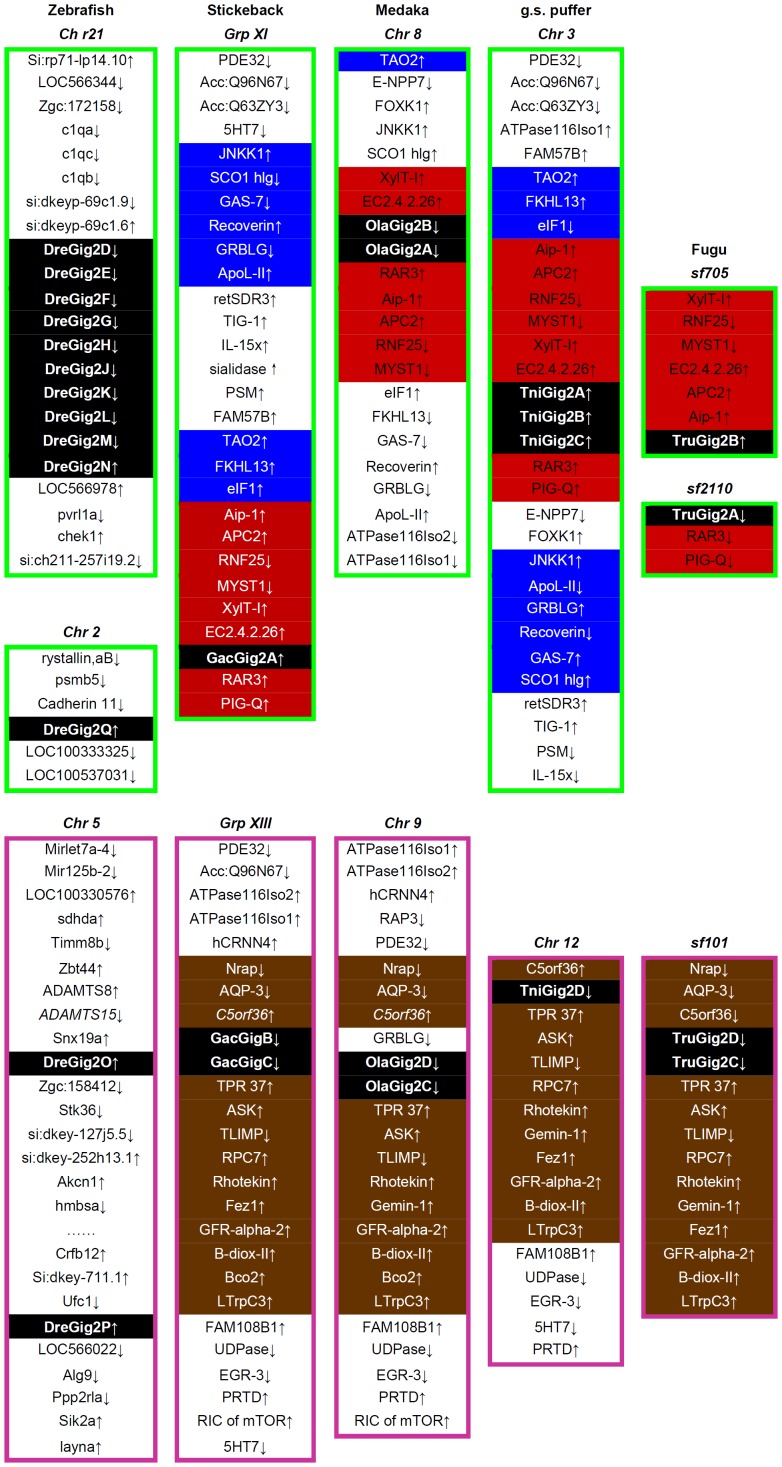
Synteny analysis of *Gig2* gene loci five fish species. Approximately 5-million-bp DNA sequences flanking *Gig2* gene loci of the indicated species were analyzed for gene colinearity. *Gig2* genes were highlighted in black background. The red and blue represented the surrounding genes that were conserved in the upper set of fish chromosomes, and the brown indicated the genes conserved in the lower set of fish chromosomes. The arrows indicate the gene transcription direction according to NCBI mapview or ensembl sequence viewer.

To further understand the evolution of *Gig2* family, we analysed the synteny of chromosomes currently available that contain *Gig2* genes including zebrafish, stickleback, medaka, green spotted pufferfish and fugu (Fig. 6). First, the gene synteny of chromosomes within species was compared. The common genes were found in the flanks of *Gig2* gene loci, such as *5HT7, Acc:Q96N67* and *PDE32* in stickleback group XI and XIII, *ATPase116Iso1, ATPase116Iso2, GRBLG* and *RAR3* in medaka chromosome 8 and chromosome 9, *TAO2* and *Acc:Q63ZY3* in green spotted pufferfish chromosome 3 and chromosome 12. Subsequently, the gene synteny of chromosomes among species was compared. It showed that stickleback group XI, medaka chromosome 8, green spotted puffer fish chromosome 3 and fugu scaffold 705 & 2110 shared high co-localization of *Gig2* gene loci, with some common surrounding genes (shown in red and blue in the upper panel in Fig. 6). In addition, a better gene synteny was shown in stickleback group XIII, medaka chromosome 9, green spotted pufferfish chromosome 12 and fugu scaffold 101, since a stretch of common genes were found in the flank of *Gig2* gene loci, with the same gene arrangement and the same transcriptional orientation (shown in brown in the lower panel in Fig. 6). Notably, the *Gig2* gene members in the upper set of chromosomes in Fig. 6 were grouped together in phylogenetic tree analysis (shown in red in Fig. 5A), while the genes from the lower set were clustered in another clade (Shown in pink in Fig. 5A).

## Discussion

In the present study, a novel gene family was identified. Since the founding gene is named *Gig2* [19] and this name is widely used in other literatures [22], [29], [30], [31], this gene family is termed *Gig2* gene family. Five crucian carp *Gig2* genes were identified by expression analyses, and most of *Gig2* family genes were in silico cloned by analyses of EST/GSS sequences derived from experimental data or NCBI databases, suggesting that these genes identified here are indeed active genes. Consistently, a proportion of family genes were further verified by analyses of the available genomes from fish species and amphibians. Large-scale search of the genome databases from invertebrates, reptiles, birds and mammals did not find any *Gig2* homologous genes. Although 5 GSSs from marine water species and 3 ESTs from algae were homologous to *Gig2* genes, it is reasonable that they might be caused by horizontal gene transfer [25]. Therefore, the data in the present study support a conclusion that *Gig2* gene family is specific to non-amniotes including lampreys, sharks, rays, ray-finned fishes and amphibians.

The results presented here also provide strong evidence for lineage-specific and species-specific expansion of *Gig2* gene family. The direct evidence is derived from phylogenetic analyses, where fish and amphibian Gig2 members are grouped into separated clades (Fig5). Subgroups of Gig2 members are formed dependently on different orders they belong to, even on different species, suggesting involvement of extensive segmental duplication in evolution of *Gig2* gene family. This notion is further verified by gene synteny analyses that there are varied gene copies in *Gig2* gene loci in different fish species (Fig. 6). In addition, domain analyses revealed that some Gig2 proteins harbour 2 or 4 basic units, each of which share a conserved N-terminus and a conserved C-terminus (Fig. 4A). These basic units exhibit a close evolutionary relationship (Fig. 4B), supporting that the multiple-unit Gig2 proteins might have arisen by domain duplication and domain shuffling, an internal duplication mechanism [32].

Gene families, especially with a large gene repertoire, usually provide invaluable information for the trace of gene and chromosome evolution history [2]. Previous studies have proposed a hypothesis that ancestral vertebrates have undergone two rounds of whole genome duplication, and later a third round of genome duplication (FSGD) occurred in a teleost fish ancestor [5], [33], [34]. The well-studied example to support this hypothesis is *Hox* gene family [35]. 4 ( = 1×2×2) canonical *Hox* gene clusters are found in most of jawed vertebrates and 8 ( = 1×2×2×2) or 7 *Hox* gene clusters are found in ray-finned fishes [35], [36], which almost perfectly fit the hypothesis. In the present study, the evolution of *Gig2* gene family might be another good example in detail to support FSGD hypothesis. The direct evidence is that there are two fish chromosome-related clades formed in phylogenetic tree analysis (the red and the pink in Fig. 5A) while only one clade is found in amphibians (the blue in Fig. 5A). Gene synteny analyses further confirmed that these members forming two fish clades are distributed in two sets of chromosomes in five fish species including zebrafish, stickleback, medaka, green spotted pufferfish and fugu (Fig. 6). Consistently, amphibians exhibit sublineage-specific clades instead of two chromosome-related clades (Fig. 5A). Recently, two duplicated copies of *Ugt2* (named *Ugt2a* and *Ugt2b*) were found to be located on two different chromosomes in stickleback and medaka [37]. Strikingly, *Ugt2b* copies are found in stickleback chromosome group XIII and medaka chromosome 9 [37], which is partially consistent with analyses of fish *Gig2* gene family. That is, fish-specific WGD occurred in a teleost ancestor has resulted in two sets of *Gig2* gene-containing chromosomes. With the radiation of teleosts, both sets of chromosomes are retained in different fish species and can be traceable. The first set includes zebrafish chromosome 21, the chromosome of stickleback group XI, medaka chromosome 8, green spotted puffer fish chromosome 3 and the chromosome of fugu scaffolds 705 & 2110, while the second set includes zebrafish chromosome 5, the chromosome of stickleback group XIII, medaka chromosome 9, green spotted puffer fish chromosome 12 and the chromosome of fugu scaffold 101(Fig. 5B and Fig. 6). Therefore, in addition to segmental duplication and internal duplication, fish-specific WGD also directly contributes to extensive expansion of fish *Gig2* gene family.

Homologous genes have evolved into two fundamentally different types: orthologs and paralogs, both of which differ in that orthologous genes result from speciation and paralogous genes from gene duplication [38]. Considering the teleost genome was subjected to a basal WGD subsequent to its divergence from mammals, it is reasonable that some *Gig2* genes of different fish species, but not all, are orthologous. However, it is hard to differentiate which *Gig2* genes are orthologous to each other based on the current phylogenetic tree, since differentiation of orthologs and paralogs often includes a reference to gene function. It has long been assumed that orthologs are functionally closer than paralogs [39], although functional similarity is not a definitive evidence for orthology. Moreover, the fact that multiple WGDs have occurred in some fish groups such as Atlantic salmon makes it much complex to address this issue. In phylogenetic analyses, besides the two major branches of *Gig2-*related genes that likely derived from the first WGD (the red and the pink in Fig. 5), two other sets are found, one from cypriniforms (the light green in Fig. 5A) and the other one from Salmoniforms (the dark green in Fig. 5A). These last two gene sets are without definite chromosome information; however, they might be also derived from WGD. According to the suggestion by John Gerlt and Patricia Babbitt [38], *Gig2* genes from different fish species are 'heterospecic' homologs, and the genes in the same fish might be 'isospecic' homologs to each other.

It is noted that there were some unexpected findings in phylogentic analyses (Fig. 5A). For example, a *Cynops pyrrhogaster* member (CpyGig2C) was clustered with a fish clade instead of an amphibian clade, and two fish members EluGig2A and OmoGig2 were found to cluster with two algae members. This is likely due to the quality of the proteins sequences used in phylogenetic tree. Since most of Gig2 sequences were in silico cloned by EST sequences, the integrity and continuity of the assembled sequences are needed for further confirmation. To accurately understanding of evolutionary relationship of *Gig2* family members, an improved phylogeneitc tree is necessary; however, it is currently hard to accomplish since this requires accurate and complete protein information that is unavailable. In addition, the gene synteny analyses did not reveal any common surrounding genes of *Gig2* gene loci between zebrafish and the other fish species (Fig. 6). The reason is likely due to genome assembly quality. In fact, there were several common surrounding genes found when gene synteny analyses were performed based on zebrafish genome databases of Zv7 (data not shown). Similarly, a total of 14 *Gig2* genes are found in zebrafish Chromosome 21 of Zv7 but only 10 in zebrafish Chromosome 21 of Zv9. Although zebrafish *DreGig2A* and *DreGig2I* are not found in zebrafish Chromosome 21 of Zv9 (Fig. 6), RT-PCR is still able to detect the transcripts of *DreGig2A* and *DreGig2I* in zebrafish tissues (Fig. 3) [23]. Despite of these issues needed to be improved, our results clearly suggest that *Gig2* gene family is unique to non-amniote vertebrates and that this gene family has been extensively expanded by lineage-specific duplication (Fig. 5B).

*Gig2* was first identified as a differentiated expressed gene in response to viral infection [19], [20], and later as a IFN-induced gene based on promoter analysis [21]. Similarly, the other four *Gig2* genes identified in crucian carp are also induced by viral infection (Fig. 1), suggesting that they might have a similar function, for example, an antiviral role as DreGig2I does [23]. Although nothing is known about their function of the conservative structures of Gig2 family proteins, domain analyses showed that they all harbour a stretch of N-terminal amino acids weakly homologous to the PARP domain of PARPs (Fig. 4B). Interestingly, a recent study showed that the PARP domain is responsible for negative regulation of viral protein by PARP1 [12]. In addition, expression profiling of zebrafish *Gig2* genes showed that at least 4 genes are not transcriptionally activated by poly(I:C), an effect IFN inducer [8]. The differential induction suggest that zebrafish *Gig2* family genes have diverged in their function with expansion of gene family, likely by some duplicates acquiring novel regulatory control, similar to a result by genome-wide comparative analyses of gene families in five teleost fish species [40].

Given the essential function of Gig2 genes under stresses, it is intriguing why this gene family is found exclusively in lower vertebrates from lampreys to amphibians. Considering that these lower organisms live in a similar environment, such as in water, half in water, or at least in very humid environments, they suffer from some common threats such as hypoxia and common pathogens, or they have some common developmental events or morphological structures. The origin of *Gig2* gene family might enable non-amniotes to adapt to the aquatic environments by evolution of lineage- or species-specific phenotypic traits. For example, extensive expansion of the *Claudin* gene family has resulted in a subgroup of fish-specific *Claudin* genes, which might be involved in regulating the exchange of specific solutes with the aqueous environment in fishes [41]. However, answering the issue of what is the physiological significance of Gig2 gene family in non-amniotes should wait for clarity of these gene function in the future studies.

## Methods

### Ethics Statement

This study was performed in strict accordance with the recommendations in the Guide for the Care and Use of Laboratory Animals of the Chinese Academy of Sciences. The protocol was approved by the Committee on the Ethics of Animal Experiments of the Institute of Hydrobiology (Permit Number: Y213191301). All surgery was performed under sodium pentobarbital anesthesia, and every effort was made to minimize suffering.

### Cells, Virus, Fish and Induction

*Carassius auratus* blastulae embryonic cells (CAB) and zebrafish liver cells (ZFL) were maintained in medium 199 supplemented with 10% fetal calf serum (FCS) at 28°C. GCRV was proliferated in CAB cells and UV inactivation of GCRV was carried out as described previously [20]. For induction, cells were seeded in 25 cm^2^ flasks one day in advance, and washed with PBS once and then treated with 0.5 ml UV-inactivated GCHV containing medium the tilters of which was 1×10^9^ TCID_50_/ml before exposing to UV irradiation. After incubation at 28°C for 1 h, the medium of treated cells were replaced with appropriate FCS-free 199 medium. The cells were collected at 2, 6, 12, 24, 48, 72, 96 and 120 h after treatment. Control cells were treated with FCS-free 199 medium alone. RNA samples of the cultured cells and fish tissues were prepared according to a previous report [20], [21]. Transfection assays were performed according to previous reports [8], [21]. Zebrafish (*Danio rerio*) were maintained at 28.5°C on a 14 h light/10 h dark cycle. All embryos used were collected by natural spawning and staged according to standard procedures [42].

### Sequence Cloning and RT-PCR

Primers were designed against the previously screened *Gig2* ESTs to PCR amplify both 5′ and 3′ termini of *CauGig2* genes by RACE from the SMART cDNA library we previously constructed [24]. The PCR products were ligated into the pMD18-T vector (Takara) and transformed into competent *E. coli* cells. The total RNA extraction and Semi-quantitative RT-PCR and Real-time PCR were performed as the previous description [20], [21]. The data by Real-time PCR were analysed by the 2^−ΔΔCT^ methods [43]. 14 pairs of specific primers were designed to specifically amplify each member of the family in zebrafish except *DreDig2B, DreGig2G, DreGig2C, DreGig2M, DreGig2R, DreGig2S,* and *DreGig2T* (Table S3). Because *DreGig2A* and *DreGig2B* have high sequence identity, it’s difficult to design specific primers to distinguish them. *DreGig2A* has a 5′UTR according to which we designed a primer specific to it. Similarly *DreGig2G* and *DreGig2H* also have high sequence identity, but *Gig2H* has a 3′UTR according to which the specific primer was designed. We failed to design primers specific to *Gig2B* and *Gig2G*. *Gig2C* and *Gig2M* were considered to be pseudogenes and were not detected. As *Gig2N* has two isoforms, we designed two pairs of primers one of which amplified both isoforms and the other of which was specific to the larger isoform. Because all members of the family have high sequence similarity, we checked all the products of RT-PCR by sequencing.

### Analysis of *Gig2* Genes in Genome Databases

The 5 crucian carp *Gig2* sequences were first subjected to search the zebrafish genome database using BLAST search against NCBI zebrafish genome database (Zv9.0). The annotated *Gig2* homologues retrieved by first search were further used as sequence baits for TBLASTN. Mapview of *Gig2* genes in zebrafish genomes was revised from the mapview provided by NCBI. The same analyses were performed in genome databases from torafugu (*Takifugu rubripes,* FUGU4), spotted green pufferfish (*Tetraodon nigroviridis,* TETRAODON8), three-spinned stickleback (*Gasterosteus aculeatus,* BROADS1), medaka (*Oryzias latipes*, MEDAKA1), and western clawed frog (*Xenopus tropicalis,* JGI 4.2).

### Sequences Survey and in Silico Cloning

Crucian carp and zebrafish *Gig2* sequences were used as query to BLAST search all the sequence databases on NCBI and ensembl. The hit sequences were downloaded and organised in a spreadsheet. The ESTs were assembled by the CAP3 online service at http://pbil.univ-lyon1.fr/cap3.php. Protein sequences of the assembled EST sequences were deduced by DNAMAN for Linux (Lynnon Corporation).

### Multiple Sequence Alignment and Phylogenetic Analysis

Multiple sequences alignments were performed by ClustalW. The alignment sequences were visualized by boxshade (http://www.ch.embnet.org/software/BOX_form.html). The evolutionary history of all the studied Gig2 protein sequences was inferred using the Neighbour-Joining method with the bootstrap test 1000 replicates in the software Geneious (http://www.geneious.com/). The evolutionary distances were computed using the Poisson correction method. All positions containing alignment gaps and missing data were eliminated only in pairwise sequence comparisons (Pairwise deletion option).

### Synteny Analysis

Synteny analysis was performed by the pipeline of SynBlast which uses the genomic region around a focal reference gene to retrieve candidates for homologous regions from a collection of target genomes and ranks them in accord with the available evidence for homology [44]. Briefly, here we used *Gig2* as a focal reference gene to retrieve candidate mRNA sequence from 5 Mbp regions at both of its flanks in each chromosome sequence. All the retrieved mRNA sequences were in turn used as query to BLAST search the chromosome sequences. The mRNA sequences which hit more than two chromosome sequences were collected in Table S4 and figured out.

## Supporting Information

Figure S1 **The common taxonomy tree was constructed using NCBI taxbrowser with 10 representative species and 52 species (in blue) where** ***Gig2*** **sequences were found.** The representative amniotes were coloured in light blue to emphasize the lack of Gig2 genes. The taxonomy IDs used in this tree are listed in the Table 1 or Table S1. The bracket immediately after the name of each species indicates the abbreviation of the species.
(PDF)

Table S1 The detailed information for all the identified *Gig2* family genes, including in silico information, cDNA sequences, ORF sequences, putative protein sequences, and the locations corresponding to putative PARP-like domains.
(XLS)

Table S2 The detailed information for 890 EST sequences homologous to *Gig2* in the study.
(XLS)

Table S3 Primers used for RACE-PCR and expression analyses in the present studies.
(DOC)

Table S4 Genes used in gene synteny analysis and corresponding accession numbers.
(DOC)
