# Supplementary material for: Identification of a Novel Gig2 Gene Family Specific to Non-Amniote Vertebrates
Source: PLoS One. 2013 Apr 4;8(4):e60588. doi: 10.1371/journal.pone.0060588 (PMC3617106; doi:10.1371/journal.pone.0060588)
Supplement: Figure S1 — The common taxonomy tree was constructed using NCBI taxbrowser with 10 representative species and 52 species (in blue) where Gig2 sequences were found. The representative amniotes were coloured in light blue to emphasize the lack of Gig2 genes. The taxonomy IDs used in this tree are listed in the Table 1 or Table S1. The bracket immediately after the name of each species indicates the abbreviation of the species. (PDF) [file pone.0060588.s001.pdf]

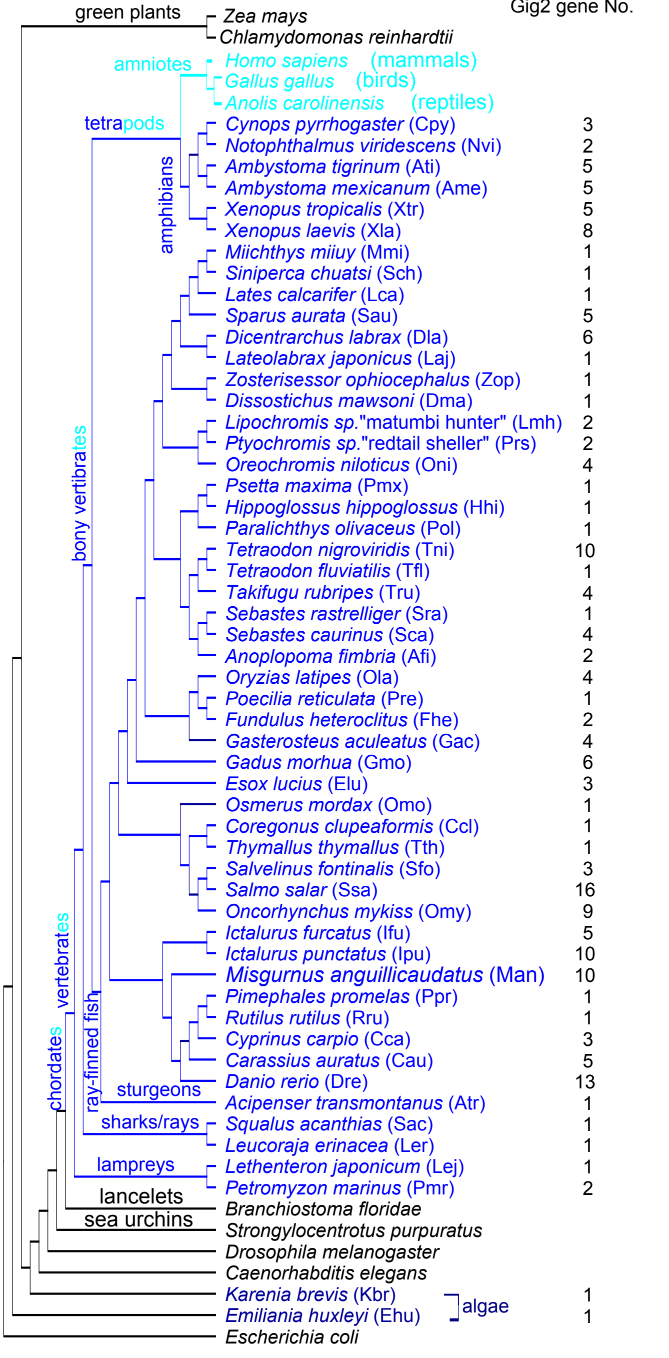
Gig2 gene No.
green plants
Zea mays
Chlamydomonas reinhardtii
amniotes
Homo sapiens (mammals)
Gallus gallus (birds)
Anolis carolinensis (reptiles)
tetrapods
amphibians
Cynops pyrrhogaster (Cpy) 3
Notophthalmus viridescens (Nvi) 2
Ambystoma tigrinum (Ati) 5
Ambystoma mexicanum (Ame) 5
Xenopus tropicalis (Xtr) 5
Xenopus laevis (Xla) 8
Miichthys miiuy (Mmi) 1
Siniperca chuatsi (Sch) 1
Lates calcarifer (Lca) 1
Sparus aurata (Sau) 5
Dicentrarchus labrax (Dla) 6
Lateolabrax japonicus (Laj) 1
Zosterisessor ophiocephalus (Zop) 1
Dissostichus mawsoni (Dma) 1
Lipochromis sp."matumbi hunter" (Lmh) 2
Ptyochromis sp."redtail sheller" (Prs) 2
Oreochromis niloticus (Oni) 4
Psetta maxima (Pmx) 1
Hippoglossus hippoglossus (Hhi) 1
Paralichthys olivaceus (Pol) 1
Tetraodon nigroviridis (Tni) 10
Tetraodon fluviatilis (Tfl) 1
Takifugu rubripes (Tru) 4
Sebastes rastrelliger (Sra) 1
Sebastes caurinus (Sca) 4
Anoplopoma fimbria (Afi) 2
Oryzias latipes (Ola) 4
Poecilia reticulata (Pre) 1
Fundulus heteroclitus (Fhe) 2
Gasterosteus aculeatus (Gac) 4
Gadus morhua (Gmo) 6
Esox lucius (Elu) 3
Osmerus mordax (Omo) 1
Coregonus clupeaformis (Ccl) 1
Thymallus thymallus (Tth) 1
Salvelinus fontinalis (Sfo) 3
Salmo salar (Ssa) 16
Oncorhynchus mykiss (Omy) 9
Ictalurus furcatus (Ifu) 5
Ictalurus punctatus (Ipu) 10
Misgurnus anguillicaudatus (Man) 10
Pimephales promelas (Ppr) 1
Rutilus rutilus (Rru) 1
Cyprinus carpio (Cca) 3
Carassius auratus (Cau) 5
Danio rerio (Dre) 13
bony vertibrates
ray-finned fish
vertebrates
chordates
sturgeons
Acipenser transmontanus (Atr) 1
sharks/rays
Squalus acanthias (Sac) 1
Leucoraja erinacea (Ler) 1
lampreys
Lethenteron japonicum (Lej) 1
Petromyzon marinus (Pmr) 2
lancelets
Branchiostoma floridae
sea urchins
Strongylocentrotus purpuratus
Drosophila melanogaster
Caenorhabditis elegans
Karenia brevis (Kbr) 1
Emiliania huxleyi (Ehu) 1
algae
Escherichia coli
