# Supplementary material for: Identification of a Novel Gig2 Gene Family Specific to Non-Amniote Vertebrates
Source: PLoS One. 2013 Apr 4;8(4):e60588. doi: 10.1371/journal.pone.0060588 (PMC3617106; doi:10.1371/journal.pone.0060588)
Supplement: Table S3 — Primers used for RACE-PCR and expression analyses in the present studies. (DOC) [file pone.0060588.s004.doc]

## Table S3 Primers used for RACE-PCR and expression analyses in the present studies.

| Primer name | Sequences (5’-3’) | Usage |
| --- | --- | --- |
| Smart-F  Smart-R | AACGCAGAGTACGCGGG  CAGAGTACT_16_ | RACE-PCR |
| CauGig2ia-F  CauGig2ia-R | AGTCTTCTAATGGGATGCTT  TTAAGAACACACACGAAAGG | Real-time PCR  RT-PCR |
| CauGig2ib-F  CauGig2ib-R | CGTCTACTAAAGGAAAACTAA  TAATACTTATCACAGACACC | RACE-PCR  Real-time PCR  RT-PCR |
| CauGig2d-F  CauGig2d-R | GACCTCTCGACATGCTGCA  AACGGTCTGAATCCGACG | RACE-PCR  Real-time PCR  RT-PCR |
| CauGig2a-F  CauGig2a-R | ACTTGACAAGCCGCATGAGA  CATCCTCCTTAGATGTGCCA | RACE-PCR  Real-time PCR  RT-PCR |
| CauGig2O-F  CauGig2O-R | TTCCGCCAAACAGCAAG  GACAAAGATCGCAGCCT | RACE-PCR  Real-time PCR  RT-PCR |
| DreGig2a-F  DreGig2a-R | TTGATGTATGATTGTGTGACC  TTGTTGATGGCTGGAACC | RT-PCR |
| DreGig2d-F  DreGig2d-R | CCCTTTCTGCACCTGTCC  CCATACCTGGTCCACCC | RT-PCR |
| DreGig2e-F  DreGig2e-R | AGGCTTTCGGGCATCTG  GGATCCTCTGGATTAATTTTGG | RT-PCR |
| DreGig2f-F  DreGig2f-R | GGTGGAGATGGGTCAGC  GACCTTGGAGGTTAATCCTC | RT-PCR |
| DreGig2h-F  DreGig2h-R | GTATGTTCTGATCCCTAGAC  GAAGTTCACAGTCCAGAAG | RT-PCR |
| DreGig2i-F  DreGig2i-R | GAAGACCAGAGAGTGATTGT  AACCATTTTCCTCTTCTTCG | RT-PCR |
| DreGig2j-F  DreGig2j-R | AGACAACCAGCGAATGG  CAGTGTCATAACGTCGCT | RT-PCR |
| DreGig2k-F  DreGig2k-R | CAGCACCAGAATATTATAAAG  CCTTGAAAACCACCAGA | RT-PCR |
| DreGig2l-F  DreGig2l-R | CTAGATACCCTGTGGAGC  ATAAGGGTCACCAAAGCC | RT-PCR |
| DreGig2n-F1  DreGig2n-R1 | ATGTTCGGGGACTTGTAG  AGAGTCCCGTGGTACAT | RT-PCR  RACE-PCR |
| DreGig2n-F2  DreGig2n-R2 | AGGTAACATTTATTCATCGC  TGCGGTTCCTTTGTGT | RT-PCR  RACE-PCR |
| DreGig2o-F  DreGig2o-R | GGCTGATTCGCAGTAAAC  CCATACACCACAATGCAGT | RT-PCR |
| DreGig2p-F  DreGig2p-R | TCACAGACAGTCACTCAG  GTAATACTTCCTCACAGGTG | RT-PCR |
| DreGig2q-F  DreGig2q-R | CGGTTATGACACTGGAATTC  CCAAAGGGCAGTGTTCT | RT-PCR |
