# Supplementary material for: Identification of a Novel Gig2 Gene Family Specific to Non-Amniote Vertebrates
Source: PLoS One. 2013 Apr 4;8(4):e60588. doi: 10.1371/journal.pone.0060588 (PMC3617106; doi:10.1371/journal.pone.0060588)
Supplement: Table S4 — Genes used in gene synteny analysis and corresponding accession numbers. (DOC) [file pone.0060588.s005.doc]

## Table 4S. Genes used in synteny analysis and corresponding accession numbers.

| Description | Access_No | Description | Access_No |
| --- | --- | --- | --- |
| 5HT7 | ENSGACT00000014575 | JNKK1 | ENSORLT00000001286 |
| Acc:Q63ZY3 | ENSTNIT00000021984 | LOC555266 | XM_677733.3 |
| Acc:Q96N67 | ENSGACT00000009160 | LOC791588 | XM_001331265.1 |
| Aip-1 | ENSORLT00000001327 | LTrpC3 | ENSORLT00000013467 |
| APC2 | ENSTNIT00000014436 | MYST1 | ENSORLT00000001404 |
| ApoL-II | ENSORLT00000001805 | Nrap | ENSORLT00000012737 |
| AQP-3 | ENSORLT00000012760 | PDE32 | ENSGACT00000009640 |
| ASK | ENSORLT00000012968 | PIG-Q | ENSTNIT00000014431 |
| ATPase116kDaIso1 | ENSORLT00000005182 | pol polyprotein | XM_001920049.1 |
| ATPase116kDaIso2 | ENSORLT00000008503 | PRTD | ENSORLT00000014067 |
| Bco2 | XM_001333649.2 | PSM | ENSTNIT00000000442 |
| B-diox-II | ENSORLT00000013333 | RAR3 | ENSORLT00000001314 |
| C5orf36 | ENSORLT00000012822 | Recoverin | ENSORLT00000001627 |
| Cav2.2 | XR_045053.1 | retSDR3 | ENSTNIT00000005007 |
| Collagen Va1 | XM_685787.3 | Rhotekin | ENSTNIT00000012976 |
| EC2.4.2.26 | ENSORLT00000001297 | RIC of mTOR | XM_001921872.1 |
| EGR-3 | ENSORLT00000013778 | RNF25 | ENSORLT00000001383 |
| eIF1 | ENSORLT00000001507 | RPC7 | ENSTNIT00000012978 |
| E-NPP7 | ENSORLT00000001120 | rystallin,aB | NM_001002670.2 |
| FAM108B1 | ENSTNIT00000013928 | SCO1 homolog | ENSTNIT00000006629 |
| FAM57B | ENSTNIT00000005950 | si:ch211-202f5.3 | XM_001333701.2 |
| Fez1 | ENSTNIT00000012973 | si:ch211-89p1.1 | NM_001082982.1 |
| FKHL13 | ENSTNIT00000014446 | sialidase | NM_001077538.1 |
| FOXK1 | ENSORLT00000001188 | TAO2 | ENSTNIT00000007064 |
| GAS-7 | ENSTNIT00000014389 | TIG-1 | ENSTNIT00000009427 |
| Gemin-1 | ENSTNIT00000012975 | TLIMP | ENSTNIT00000012981 |
| GFR-alpha-2 | ENSORLT00000013248 | TPR 37 | ENSORLT00000012936 |
| Gig2 | XM_001340877.1 | UDPase | ENSORLT00000013672 |
| GRBLG | ENSORLT00000001730 | XylT-I | ENSTNIT00000014433 |
| hCRNN4 | ENSORLT00000008817 | zgc:100952 | NM_001003781.1 |
| HMBS synthase a | NM_201154.1 | zgc:174263 | NM_001114314.1 |
| IL-15x | ENSTNIT00000004122 |  |  |
